# Poly (ADP-Ribose) Polymerase inhibitors (PARPi) therapy response in an acral melanoma patient with EMSY gene amplification

**DOI:** 10.1016/j.jdcr.2024.04.005

**Published:** 2024-04-16

**Authors:** George Nassief, Omar H. Butt, Alice Y. Zhou, George Ansstas

**Affiliations:** Division of Medical Oncology, Department of Medicine, Washington University in Saint Louis, Saint Louis, Missouri

**Keywords:** acral melanoma, *EMSY*, immunotherapy resistance, PARPi

## Introduction

Poly (ADP-Ribose) Polymerase inhibitors (PARPi) have been implicated in recent years as viable treatment options for melanoma patients with homologous recombination deficiency (HRD) status. However, patient selection for PARPi therapy remains challenging as there remains many genetic and epigenetic factors that could contribute to HRD status.

Here, we report a case of an advanced acral melanoma patient, a more aggressive form of melanoma, with disease progression following standard-of-care treatment who demonstrated a near-complete response to PARPi treatment.

## Case report

An 88-year-old male with a history of atrial fibrillation, multiple squamous and basal cell carcinoma on arms and back status postexcision, and a history of stage IIB acral melanoma presents with recurring melanoma. His initial stage IIB (pT3bN0M0) acral melanoma was treated with a toe amputation and lymph node biopsy before starting surveillance 6 years ago. Two years postsurgery, he developed biopsy-proven in-transit lesions on the left shin. PET-CT showed hypermetabolic lesions along the left lower extremity and mediastinal lymph nodes. For the mediastinal lymphadenopathy, endobronchial ultrasound and fine needle biopsy of the subcarinal, right hilar, and right paratracheal lymph nodes were performed and were negative for signs of metastatic melanoma. Therefore, he underwent wide local excision of the in-transit lesions.

Subsequently, he received 11 months of adjuvant pembrolizumab, itself complicated by grade 2 colitis requiring steroids. One year following immunotherapy, restaging PET-CT showed 2 hypermetabolic abnormalities in the left calf, which on biopsy demonstrated recurrent melanoma. Next-generation sequencing was conducted on a biopsy from an in-transit lesion on the left lateral mid-calf using the Tempus xT assay (Tempus AI, Inc). Briefly, Tempus xT is a targeted, tumor-normal-matched DNA panel that detects single-nucleotide variants, insertions and/or deletions, and copy number variants in 596-648 genes, as well as chromosomal rearrangements in 22 genes with high sensitivity and specificity. Molecular profiling identified somatic copy number gain of ≥20 for *EMSY* and 13 for *CCND1*, *PAK1*, and *RSF1* (threshold for amplification ≥8 copies), along with a Genome-Wide Loss of Heterozygosity score of 20.9% (threshold ≥33%). There were no corresponding potential germline mutations in the matched blood sample.

In-transit lesions following immunotherapy prompted repeat surgery and local radiotherapy of 5000 cGy in 20 fractions over 4 weeks. One-year postradiotherapy, he developed in-transit progression prompting intra-tumoral injections (ITI) of oncolytic talimogene laherparepvec virus given for 3 weeks between the first and second treatment and every 2 weeks following that with initial clinical response. Restaging PET-CT scans 7 months after ITI revealed hypermetabolic lymphadenopathy/soft tissue nodules including peripancreatic, left inguinal, and left popliteal hypermetabolic lesions consistent with systemic metastatic progression. ITI therapy was stopped.

**Fig 1:**
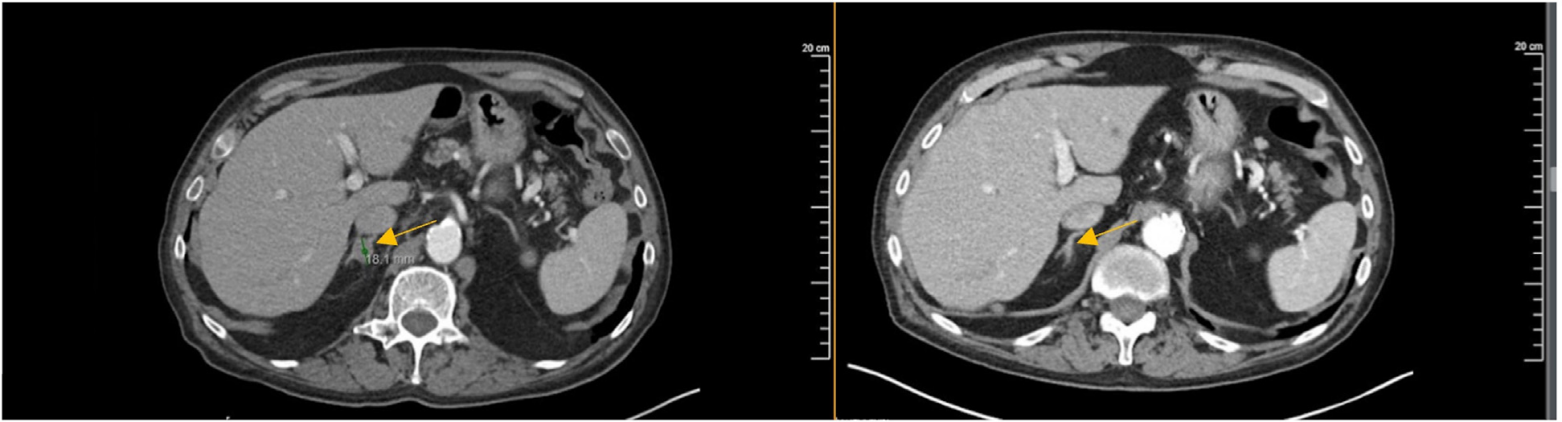
CT scan of an adrenal nodule before and after PARPi treatment. CT scan (*left* scan) taken before PARPi therapy course showing a 1.8 cm adrenal nodule, which demonstrated reduction following treatment (*right* scan). *Yellow arrow* pinpoints the adrenal nodule before and after therapy.

Two months of temozolomide was initiated, however, no meaningful response was obtained. This prompted the initiation of talazoparib at 1 mg daily. After 13 months of talazoparib, a near-complete response was observed (Fig 1). Talazoparib treatment was complicated by anemia, which resolved with a drug holiday and dose reduction to 0.75 mg daily. At the time of this report, the patient has been on talazoparib for 14 months with a sustained response.

## Discussion

To our knowledge, this is the first case report of a metastatic acral melanoma patient with *EMSY* gene amplification, without abnormal *BRCA2* expression levels, to achieve a near-complete response to the PARPi talazoparib.

Homologous recombination DNA damage (HR-DDR) pathway gene mutations are known to be biomarkers for PARPi response. However, several studies have identified patients without traditional HR-DDR gene mutations that respond to PARPi therapy, underscoring the need to explore potential genetic and epigenetic markers outside of HR-DDR pathway gene mutations.^1^^,^^2^

It has been previously proposed that *EMSY* amplification could lead to increased expression of the C11orf30 protein, a nuclear protein. The overexpression of this protein has been shown to have a *BRCA2* transactivation potential, induce chromosomal instability, and mimic the activity of *BRCA2* mutations in different types of cancers including breast and ovarian cancers.^3^ Ihnen et al identified that cell lines with *EMSY* amplification were associated with sensitivity to rucaparib, a PARPi treatment, and that the treatment showed therapeutic advantage in sporadic ovarian patients with HRD status.^4^ However, Wilkerson et al also identified that cell lines from distinct tumor sites including the breast, ovary, pancreas, esophagus, lung, and oral cavity with *EMSY* gene amplification did not demonstrate significantly different sensitivity to HR-targeted therapies when compared to cell lines without the gene amplification.^5^ The conflicting preclinical data available thus call the need for more research into the viability of *EMSY* gene amplification as a predictive biomarker for PARPi response, especially in malignant melanoma cells due to the novel response demonstrated in this report.

The *EMSY* gene was amplified along with *CCND1*, *PAK1*, and *RSF1* most likely due to these genes’ grouped location within the 11q13 amplicon region.^6^ Although the genes amplified, like *CCND1*, *PAK1*, and *RSF1*, they have been implicated as potential oncogenes, *EMSY* and *CCND1,* have been proposed as the most likely drivers of oncogenesis.^6^^,^^7^ Due to the amplification of other potential oncogenes, we cannot confirm that the *EMSY* gene amplification is the response marker for PARPi in this report. However, its putative activity within the HR-DDR pathway and its interaction with the *BRCA2* gene, an already established marker for PARPi response, argues for its viability as a predictor for response.

The cBioPortal was assessed for patient samples that tracked *EMSY* gene amplification in melanoma. In a 144 metastatic melanoma patient sample queried by cBioPortal, 2.78% demonstrated *EMSY* amplification.^8^^,^^9^ In another sample of 64 melanoma patients, 6.25% also had *EMSY* amplification.^8^^,^^10^ Although these datasets are limited by the sample size, *EMSY* gene amplification is prevalent in melanoma. Given the limited treatment options for acral melanoma, which has lower responses to standard-of-care immunotherapies than cutaneous melanoma, finding novel biomarkers to select patients for possible treatment is clinically significant.

Our case highlights the importance of investigating the potential usage of *EMSY* gene amplification as a response marker to PARPi therapy. However, it is important to recognize that this is a case of one patient and further investigations are needed to test the efficacy and safety of PARPi treatment in this patient population.

## Conflicts of interest

None disclosed.
